# Synthesis and characterization of a hyper-branched water-soluble β-cyclodextrin polymer

**DOI:** 10.3762/bjoc.10.271

**Published:** 2014-11-06

**Authors:** Francesco Trotta, Fabrizio Caldera, Roberta Cavalli, Andrea Mele, Carlo Punta, Lucio Melone, Franca Castiglione, Barbara Rossi, Monica Ferro, Vincenza Crupi, Domenico Majolino, Valentina Venuti, Dominique Scalarone

**Affiliations:** 1 Dipartimento di Chimica, Università di Torino, Via Pietro Giuria 7, 10125 Torino, Italy; 2 Dipartimento di Scienza e Tecnologia del Farmaco, Università di Torino, Via Pietro Giuria 9, 10125 Torino, Italy; 3 Department of Chemistry, Materials and Chemical Engineering “G. Natta”, Politecnico di Milano, Piazza L. da Vinci 32, 20132 Milano, Italy; 4 Elettra - Sincrotrone Trieste. Strada Statale 14 km 163.5, Area Science Park, 34149 Trieste, Italy and Department of Physics, University of Trento, Via Sommarive 14, 38123 Povo, Italy; 5 Dipartimento di Fisica e Scienza della Terra, Università di Messina, Viale F. Stagno d'Alcontres 31 Contrada Papardo, 98166 Messina, Italy

**Keywords:** β-cyclodextrin, complexation, gelation, polyelectrolytes, soluble β-cyclodextrin polymers

## Abstract

A new hyper-branched water-soluble polymer was synthesized by reacting β-cyclodextrin with pyromellitic dianhydride beyond the critical conditions that allow the phenomenon of gelation to occur. The molar ratio between the monomers is a crucial parameter that rules the gelation process. Nevertheless, the concentration of monomers in the solvent phase plays a key role as well. Hyper-branched β-cyclodextrin-based polymers were obtained performing the syntheses with excess of solvent and cross-linking agent, and the conditions for critical dilution were determined experimentally. A hyper-branched polymer with very high water solubility was obtained and fully characterized both as for its chemical structure and for its capability to encapsulate substances. Fluorescein was used as probe molecule to test the complexation properties of the new material.

## Introduction

Cyclodextrins (CDs) are biomolecules consisting of glucopyranoside units linked 1,4 to form cyclic oligossacharides. CDs composed of 6, 7 or 8 sugar units, which are known as α-, β- and γ-cyclodextrins respectively, are characterized by a toroidal structure with an arrangement of secondary and primary hydroxy groups that makes the inner surface of the toroid lipophilic and the outer surface highly hydrophobic. This peculiar structure allows CDs to efficiently form molecular inclusion complexes with various organic and inorganic guest compounds. Complexation properties and water solubility make CDs very interesting materials especially for pharmaceutical applications, due to their capability to solubilize lipophilic drugs, but also for environmental remediation and as supramolecular carriers in a number of synthetic procedures ranging from organometallic reactions [1] to emulsion polymerization [2–4].

By reacting CDs with suitable crosslinking agents, nanostructured hyper-cross-linked materials, known as nanosponges, can be obtained [5–7]. Currently there is a growing interest in the synthesis of soluble β-CD-based polymers, which is a demanding task considering the high number of functional groups per CD unit. To synthesize soluble CD polymers different routes can be followed: 1) grafting of CDs on pre-synthesized polymers [8] 2) polycondensation of difunctionalized CD monomers obtained by selective modification of the CD secondary hydroxy groups [9] 3) inhibition of the gelation process in a sol–gel cross-linking reaction [10]. This last synthetic route leads to soluble hyper-branched polymers. According to Flory’s theory [11] gelation occurs when the branching coefficient (α) is higher than a critical value (α_c_) defined by the following equation:

[1]
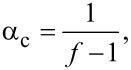


where *f* is the number of functional groups in the polyfunctional monomer. For β-CDs, α_c_ is 0.05, meaning that the branching coefficient must be kept lower than 0.05 in order to avoid gelation.

In simple systems involving a trifunctional and a bifunctional monomer the branching coefficient can be determined experimentally by assuming equal reactivity of the functional groups and using the equation below:

[2]
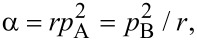


where *p*_A_ (or *p*_B_) is the probability that an A (or B) group has reacted, i.e., the extent of reaction, and is the ratio of A to B groups, where A represents the polyfunctional monomer and B the bifunctional one. At the gelation point *p* = *p*_c_ and *c* = *c*_c_, which means that gelation can be prevented by stopping the reaction after a short time, when *p* < *p*_c_. However, in the case of complex polyfunctional monomers, such as CDs, the critical branching coefficient differs significantly from the predictable one mainly because of the different reactivity of functional groups. Moreover, Flory’s theory does not take into account either the influence of the amount of solvent (critical dilution) or the frequency of intramolecular cyclization.

Different models have been developed to predict critical dilution conditions [12–13] but they apply only to simple systems, while for sol–gel processes involving polyfunctional monomers reliable conditions for critical dilution are more easily determined experimentally.

In this contribution, we describe the one step synthesis of a hyper-branched water soluble β-CD-based polymer obtained by reacting β-CD with pyromellitic dianhydride. The synthesis was performed by using an excess of solvent and a suitable cross-linker/β-CD ratio, and the conditions for critical dilution were determined. The branched structure and the molecular weight of this β-CD-based polymer were assessed by solubility tests, ultrafiltration experiments, size exclusion chromatography and Raman or FTIR spectroscopy. The new water soluble hyper-branched polymer shows complexing properties towards organic molecules and inorganic cations.

## Results and Discussion

### Synthesis and chemical characterization

**Figure 1:**
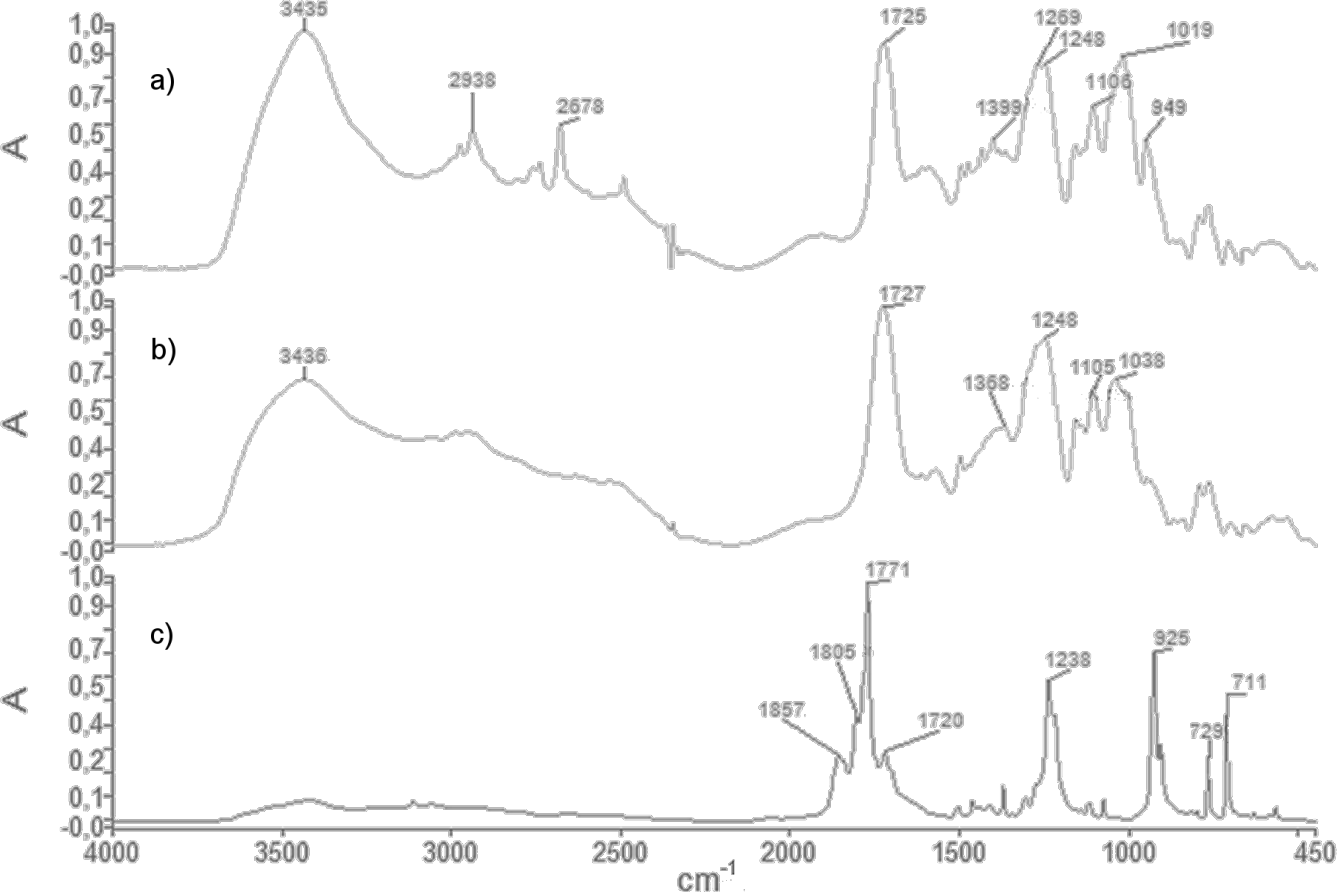
FTIR spectra of branched β-CD polymer (a), cross-linked β-CD nanosponge (b) and pyromellitic dianhydride (c).

Syntheses of soluble CD containing polymers is a hard challenge due to the great number of reactive groups located on the CD molecule that may be involved in side reactions leading to heavily cross-linked polymers. Quenching of the reaction before reaching the gelation point is one of the most used routes to get soluble CD polymers [14]. However, during polymerization the viscosity of the solution increases rapidly, thus leading to broad molecular weight distributions and poor reproducibility. A reduction in the concentration of the reactants leads to low reaction rates, but invariably, gelation occurs. The same happens by using a lower amount of catalyst, if any. Nevertheless, many papers have been published regarding the so called CD nanosponges that are highly cross-linked CD polymers [15–16]. Recently a detailed investigation of these novel nanomaterials allowed to clarify some structural aspects and functional properties, and in particular pyromellitic nanosponges were found to present a critical cross-linking value [17]. In other words, the maximum number of cross-linkers per monomer was proved to be around 6 (i.e., 6 mol of pyromellitic dianhydride for each mol of β-CD). If the amount of cross-linker is higher than this value the cross-linking agent is grafted to the CD polymer only from one side, thus leading to an increase of branching groups on the nanosponge structure. Now we have found that by working under limited dilution conditions and with a high cross-linker/CD ratio (e.g., 12:1 molar ratio), the viscosity of the resulting CD-based polymer does not change for many hours, thus proving the stability of the formed branched polymer. The reaction is carried out at room temperature and followed by relative viscosity measurements. TLC analyses confirm the consumption of pristine CD quite immediately. The occurred polymerization reaction is also confirmed by FTIR analyses (Figure 1): the absorption band of the carbonyl groups (C=O stretching) of pyromellitic dianhydride, observed at 1771 cm^−1^, is shifted to 1725 cm^−1^ in the spectrum of the branched β-CD polymer proving the ring opening reaction of the cyclic dianhydride and the formation of ester bridges. Moreover, the relative intensity of the O–H stretching band (centered at 3435 cm^−1^) with respect to the intensity of the carbonyl stretching band is higher in the branched polymer than in the cross-linked nanosponge, that is because the branched polymer contains an higher number of OH groups that under different, less controlled reaction conditions would have reacted with pyromellitic dianhydride to give ester cross-links and an hyper-cross-linked nanosponge. The O–H stretching band can be ascribed to both primary and secondary CD hydroxy groups and to carboxylic OH stretching deriving from the opening of the cyclic dianhydride. Related to this last absorption are also the bands at approximately 2500–2700 cm^−1^ that have been assigned to overtones and combinations of bands at 1300–1440 cm^−1^ due to OH bending and C–O stretching of unreacted carboxy groups. As discussed later, deconvolution of the carbonyl stretching band at 1725 cm^−1^ also shows the contribution of carboxylic acid groups in addition to ester ones.

The synthetized branched β-CD polymer has an exceptionally high solubility in water (i.e., 800 mg/mL) over a wide pH range. However, at a pH below 1 the protonation of carboxylic acid groups leads to a precipitate. As predictable, if neutral pH is restored, all the precipitated polymer is solubilized again. This behavior could be of interest in pH responsive drug delivery systems.

In addition to water the branched β-CD-based polymer is also soluble in some aprotic organic solvents such as DMF and DMSO, while it is insoluble in less polar solvents such as ethanol, acetone, ethyl ether and ethyl acetate.

Once solubilized in water, the branched β-CD polymer is able to precipitate in the presence of Cu^2+^, Fe^3+^, Ba^2+^, Cd^2+^, Pb^2+^ and Sn^2+^, by forming metal–organic complexes. Conversely, no precipitation occurs with Ca^2+^, Mg^2+^, Fe^2+^, Mn^2+^, Ni^2+^ and Ce^4+^, showing some selectivity towards certain metal cations and indirect evidence of polyelectrolyte behavior.

**Figure 2:**
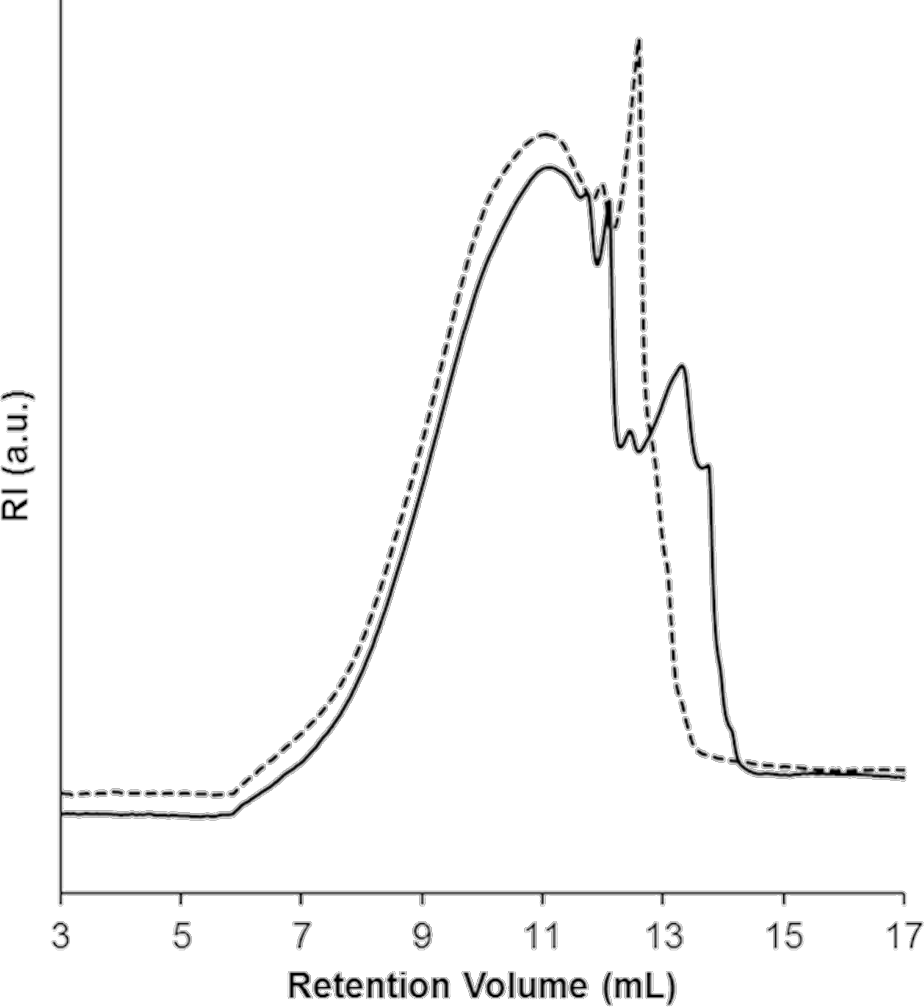
SEC curves of the β-CD-based polymer before (solid line) and after ultrafiltration with cut-off size of 30000 Da (dashed line).

A gross evaluation of the molecular weight of the branched β-CD polymer was performed by means of ultrafiltration with three different cut-off sizes: 3000 Da, 10000 Da and 30000 Da. The experiments show that less than 5% of the sample has a molecular weight lower than 3000 Da, 48% is comprised between 3000 and 10000 Da, only 3.5% is between 10000 and 30000 Da and 43.5% has a molecular weight higher than 30000 Da. These data were confirmed by size exclusion chromatography (SEC) analyses. Figure 2 shows SEC curves of the whole polymer and of the fraction of sample resulting from ultrafiltration with cut-off size of 30000 Da. The SEC curve of the CD-based polymer has a multimodal profile showing three main fractions centered at approximately 11.1 mL, 12.1 mL and 13.3 mL, and three shoulders at 11.7 mL, 12.5 mL and 13.8 mL. The filtered sample has a narrower distribution and a simpler profile, showing three main fractions centered at 11.1 mL, 12.00 mL and 12.6 mL. As expected, ultrafiltration has removed a low molecular weight fraction from the sample which is eluted between 12.8 and 14.3 mL. Average molecular weights calculated within this range of retention volumes are *M*_n_ = 37000 and *M*_w_ = 42000, both in good accordance with the employed cut-off size. Even if the applied calibration curve does not allow an accurate determination of molecular weights, but only relative molecular weight values, SEC curves confirm that most of polymer chains have a large hydrodynamic volume, corresponding to molecular weights considerably higher than 30000.

**Figure 3:**
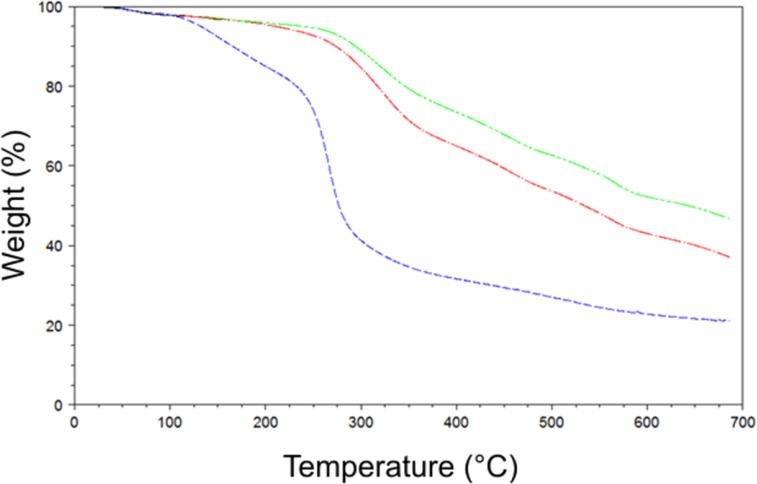
Thermogravimetric curves of the β-CD-based polymer after ultrafiltration with cut-off size of 3000 Da (blue line), 10000 Da (red line) and 30000 Da (green line).

Thermogravimetric analyses (Figure 3) clearly show that thermal stability increases by selecting molecular weights greater than 10000 Da (red line) and 30000 Da (green line), and these samples are characterized by a remarkable thermal stability. Degradation of such larger fractions starts at approximately 250 °C and proceeds more slowly than for the whole polymer, leading to a residue of ~45% at 700 °C.

Altogether these data confirm that a high molecular weight β-CD-based polymer was successfully synthetized. Furthermore, the number of functionalities carried by the starting monomers and the high cross-linker/β-CD molar ratio suggest that these water-soluble polymers are more likely composed of hyper-branched structures, rather than linear chains. This is consistent with the infrared and Raman analysis discussed below.

**Figure 4:**
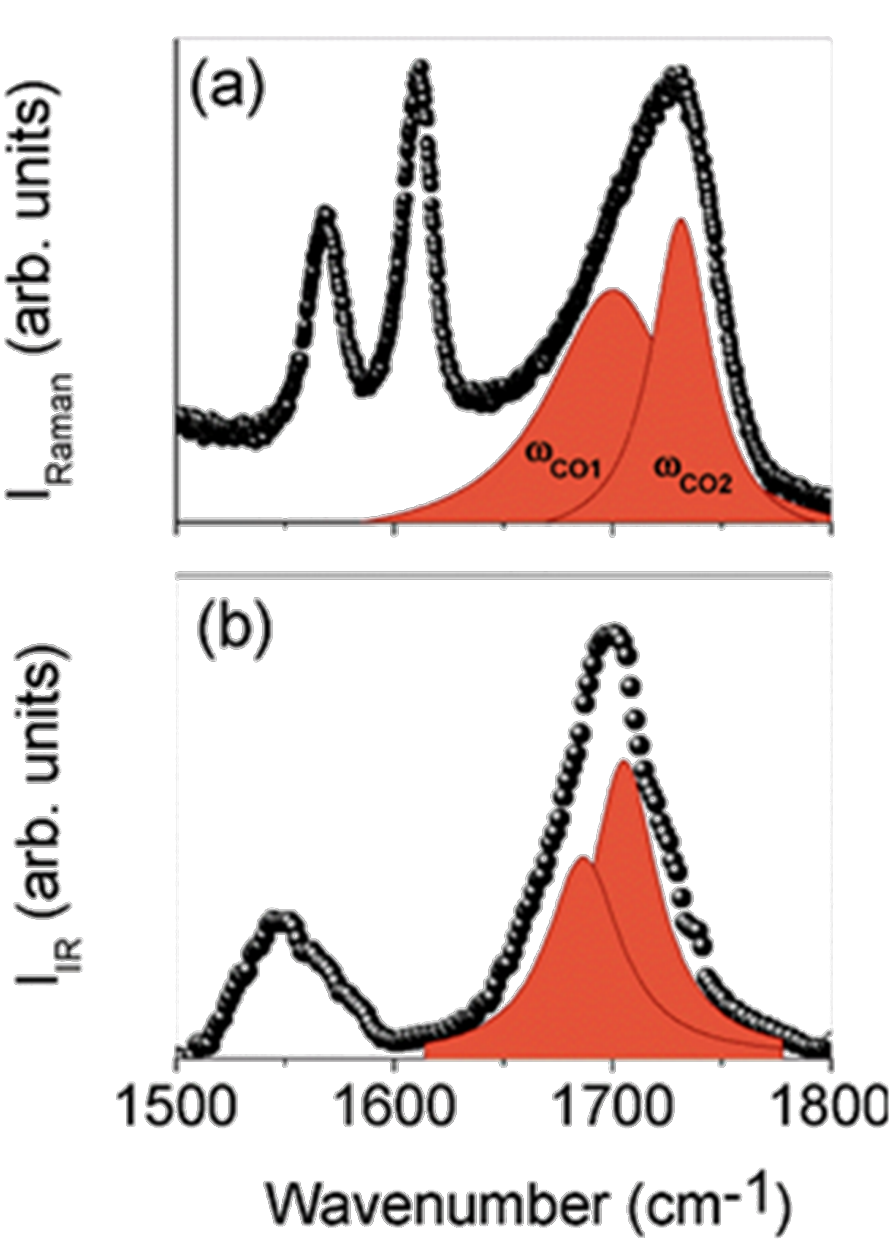
Raman (a) and FTIR–ATR (b) spectra of the branched β-CD-based polymer in the wavenumber range of 1500–1800 cm^−1^, together with the deconvolution components of the C=O stretching band.

In Figure 4a,b the Raman and FTIR–ATR spectra of the branched β-CD-based polymer are reported in the wavenumber range of 1500–1800 cm^−1^. Both spectra show an asymmetric absorption centered at 1700–1740 cm^−1^ due to the stretching vibration of carbonyl groups. According to an already successfully applied curve fitting procedure [18–19], the deconvolution of the carbonyl stretching vibration band was performed disclosing the presence of two superimposed sub-bands that have been ascribed to the vibrations of the C=O belonging to the ester groups (ω_CO1_) and to the free carboxylic groups (ω_CO2_) of the branched β-CD polymer.

**Figure 5:**
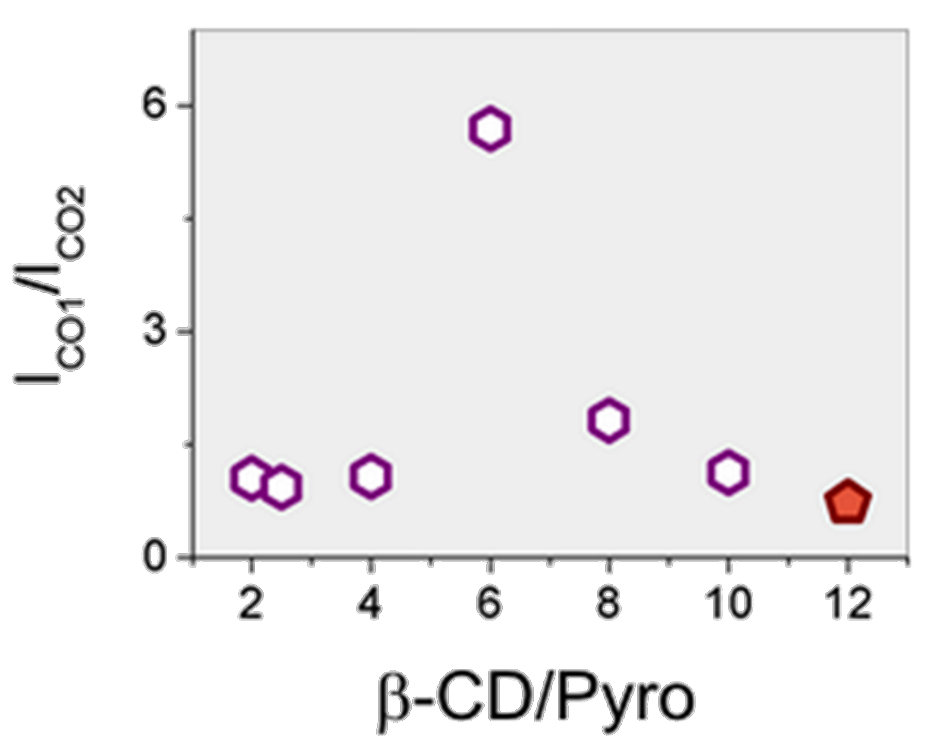
Ratio between the intensity of the bands assigned to ester groups (I_CO1_) and to the free carboxylic groups (I_CO2_) for cross-linked β-CD nanosponges (hexagons) and the β-CD-based polymer (pentagon). β-CD/Pyro = β-CD/pyromellitic dianhydride molar ratio.

After a suitable normalization of the vibrational spectra, the total estimated intensity for the C=O stretching band can be conveniently used as a semi-quantitative descriptor of the population of the C=O functional groups, as previously reported [18–21]. Actually, deconvolution of Raman spectra allows the assignment of the ester and carboxylic bands in the range of 1650–1750 cm^−1^. The soluble branched β-CD polymer presents a higher amount of total C=O groups (ester + carboxyl) with respect to the insoluble β-CD nanosponges [18–19]. It is likely that the amount of ester groups can be assumed as an index of the degree of cross-linking, whereas the amount of carboxyl groups is more related to the degree of branching. As branched β-CD polymers show a lower I_CO1_(ester)/I_CO2_(carboxyl) ratio than cross-linked β-CD nanosponges (Figure 5), this is in accordance with a hyper-branched polymer structure.

### Complexation properties

**Figure 6:**
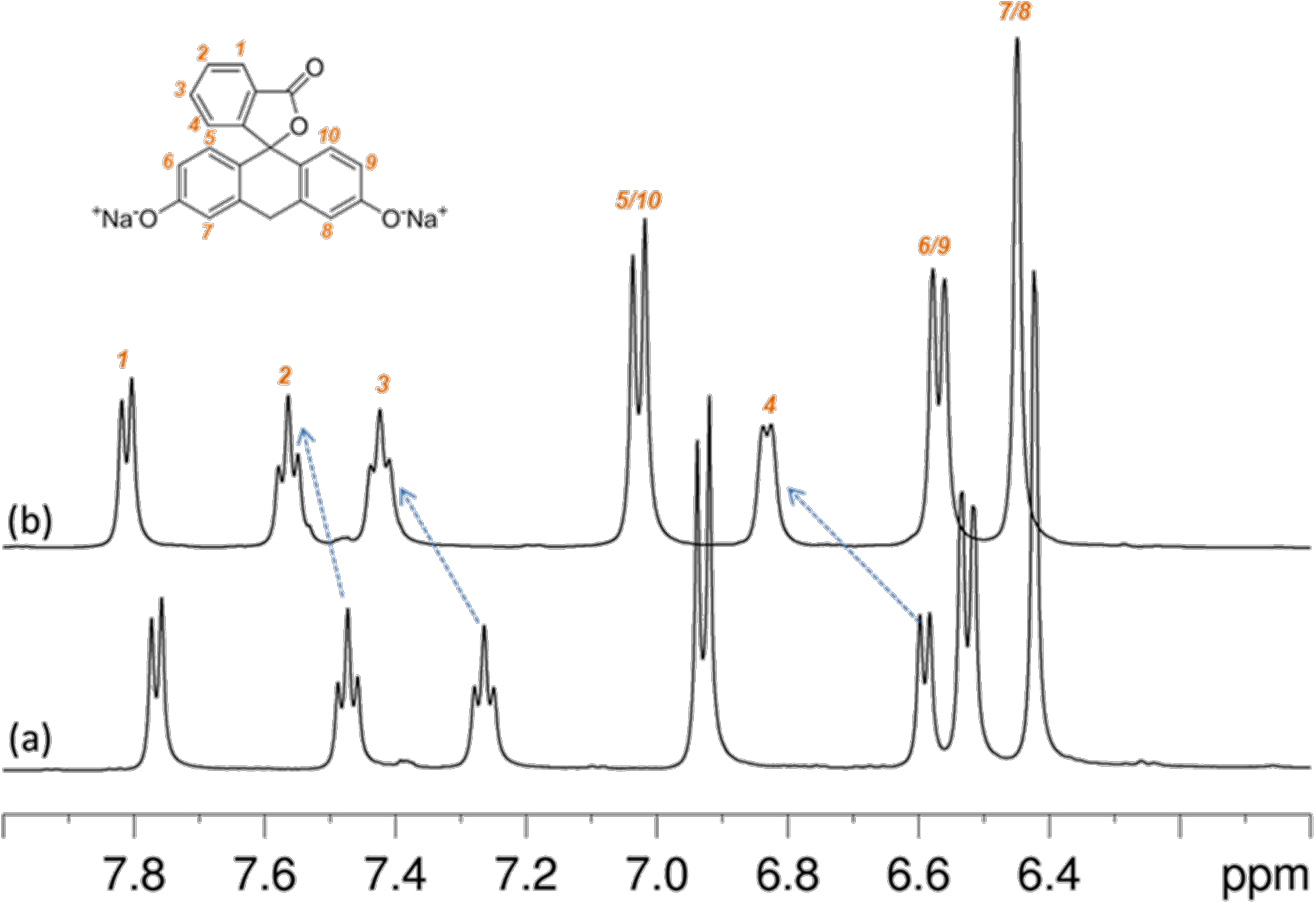
NMR spectra of fluorescein in D_2_O solution (a) and in the presence of the hyper-branched β-CD polymer (b). pH range: 8.25–7.50.

The ability of the hyper-branched β-CD polymer to form non-covalent adducts with organic molecules was tested by using sodium fluorescein (SF) as a probe molecule. The choice of this probe is due to previous use in the characterization of CD nanosponges [21] and to the presence of a strong chromofore for UV analysis. As a first attempt to test the interaction of the polymer with the substrate, we carried out ^1^H NMR measurements with D_2_O as solvent. We compared the spectra of SF in D_2_O (5 mM solution) as a reference and a second solution of SF at the same concentration but in the presence of the branched β-CD polymer (20 mg of polymer/600 μL of 5 mM SF in D_2_O). As a general remark, the ^1^H NMR spectrum of the SF/polymer mixture showed broad signals of the polymer and sharp, high resolution signals of the guest SF. This means that the association phenomena, if present, are in fast exchange on the NMR time scale. Figure 6 shows the low field expansion of ^1^H NMR spectra of 5 mM SF in D_2_O (lower trace) and in the presence of the branched β-CD polymer (upper trace). Additionally, the molecular formula and atom numbering of SF are also reported. The simple visual inspection reveals that SF protons undergo selective de-shielding in the presence of the polymer, consistent with the formation of a host–guest association. The analysis of the polymer induced chemical shift variations on SF (Δδ values, Table 1) points out that the largest de-shielding is experienced by the protons of the benzofuranone ring system, H3 and H4 in particular. This finding suggests that this fragment of SF is the major site of interaction with the branched β-CD polymer, possibly via formation of a real inclusion complex with the β-CD cavity. More detailed NMR analysis are in progress and will be reported elsewhere.

**Table 1:** ^1^H NMR chemical shifts (ppm) of sodium fluorescein (SF) in D_2_O solution without and with branched β-CD polymer. The numbers refer to SF atom numbering, symbols in parentheses indicate signal multiplicity.^a^

Sample	1 (d)	2 (t)	3 (t)	5/10 (d)	4 (d)	6/9 (d)	7/8 (d)

SF	7.77	7.47	7.26	6.93	6.59	6.53	6.42
SF in β-CD-polymer	7.81	7.56	7.42	7.03	6.83	6.57	6.45
Δδ	0.04	0.09	0.16	0.13	0.24	0.04	0.03

^a^pH range: 8.25–7.50.

**Figure 7:**
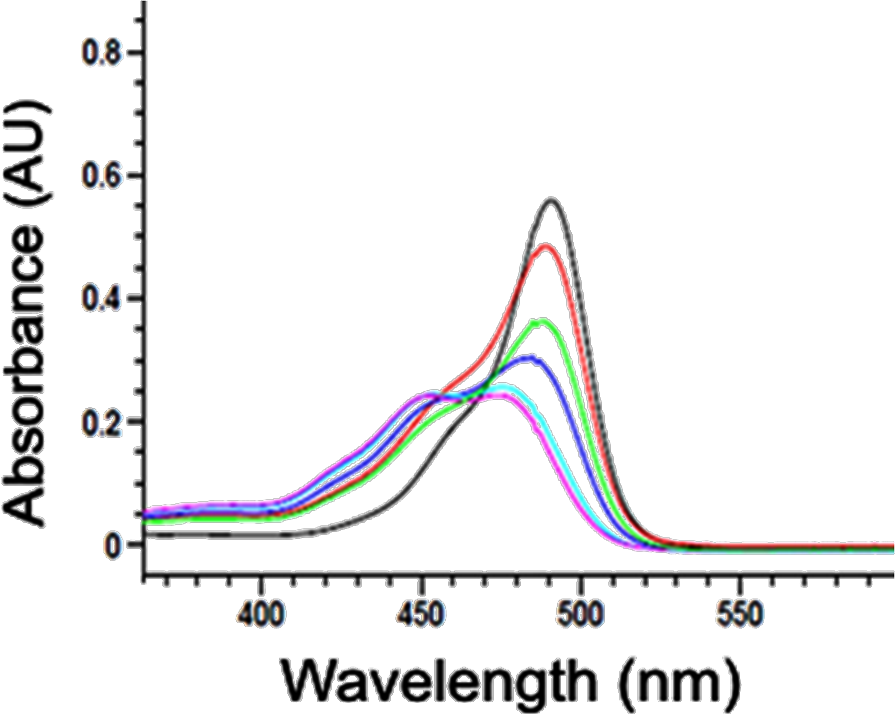
UV–vis spectrum of fluorescein with increasing amounts of hyper-branched β-CD polymer. pH range: 8.4–7.2.

The formation of inclusion complexes between SF and the branched β-CD polymer was further supported by UV–vis spectroscopy. The UV spectra of SF in the absence and presence of increasing amounts of polymer are shown in Figure 7. A decrease of absorbance was observed for the UV band of FS (490 nm) and an increase of absorbance of a new band at 450 nm, consistent with the formation of a SF/polymer complex.

The solubilization capacity of the new branched β-CD polymer was also evaluated toward a very poor water soluble anticancer drug, i.e., paclitaxel, showing a great improvement in dispersion in water (data not shown).

## Conclusion

A new hyper-branched β-CD polymer was synthetized and characterized. The synthetic route proposed seems to be quite independent from the reaction time. The new polymer exhibits an exceptionally high solubility in water, DMF and DMSO, while it is insoluble in low polar solvents and at very low pH. The molecular weight by ultrafiltration separation and SEC is estimated to be greater than 30000 Da. The polymer shows good complexing properties towards organic molecules. In addition, it strongly interacts with several metal cations.

## Experimental

β-CD was a gift of Roquette Italia (Cassano Spinola, Italy) and was dried in oven at 120 °C up to constant weight before use. Pyromellitic dianhydride, DMF and other reagents and reactants were purchased from Sigma-Aldrich and used as received.

The hyper-branched β-CD-based polymer was synthetized by dissolving 0.977 g of anhydrous β-CD in 6 mL of DMSO. To this solution (1.44 × 10^−3^ M), 1 mL of triethylamine (TEA) as catalyst was added. Then, 2.254 g (1.48 M) of pyromellitic dianhydride was added under vigorous stirring for 3 hours at room temperature. The addition of TEA had no effect on the temperature of the reaction mixture. A slight increase of the temperature was observed after the introduction of pyromellitic dianhydride. The viscosity of the solution gradually increases, but after few hours it remains constant. At this point, the solution was precipitated into an excess of diethyl ether, filtered under vacuum, solubilized in deionized water, lyophilized and finally stored in a desiccator. Obtained 3.57 g. Yield 90% (calculated by considering the weight of the polymer collected after lyophilization with respect to the expected, theoretical weight, defined by the sum of reagents, β-CD and pyromellitic dianhydride). The NMR spectrum of the polymer shows only a small, sharp peak assignable to residual DMSO. The intensity of the DMSO peak is of the same order of magnitude of the ^13^C satellites of the triethylammonium signals, the positive counterions of the soluble polymer. The amount of unreacted monomers was negligible, as clearly observed from TLC analysis and ultrafiltration essays.

Relative viscosity measurements were performed by using Ubbelohde type viscometer thermostated at 30 °C diluting 25 times the reaction solution with water.

Thin-layer chromatography was performed on Fluka 5 × 10 cm silica-coated aluminum foils with fluorescent indicator 254 nm, purchased from Sigma-Aldrich. The eluent phase was composed of deionized water, isopropanol, ethyl acetate and ammonium hydroxide (3:5:1:1 by volume). β-CD and pyromellitic dianhydride were used as references. Stains were emphasized by immersing the foils for a few seconds in a 5% H_2_SO_4_ solution in ethanol. Then, they were left to dry in air and finally heated on a hot plate.

Ultrafiltration was conducted employing cellulose membrane discs with three different cut-off sizes: 3, 10 and 30 kDa. The 3 kDa membrane discs were purchased from Millipore, Bedford, USA, whereas the 10 and 30 kDa membrane discs were provided by Generon Ltd, Maidenhead, UK. 200 mg of the soluble polymer was dissolved in 50 mL of deionized water and ultrafiltrated. Afterwards, the separated fractions of polymer were recovered by lyophilization and weighed.

Size exclusion chromatography was performed with a Viscotek modular instrument equipped with a VE 1122 pump, a VE 7510 degasser, manual injection valve, VE 3580 refractive index detector, column oven and two PLgel 10 µm MIXED-B columns (Polymer Laboratories, UK). *N*,*N*-Dimethylformamide (1.0 mL min^−1^) was used as eluent and analyses were performed setting the column oven at 70 °C. DMF solutions of the samples (3 mg/mL) were filtered through 0.45 μm PTFE membrane filters. Calibration was obtained with PEG/PEO molecular weight standards.

All Raman measurements were carried out on dried samples deposited on a glass slide in air and at room temperature. Spectra were collected in backscattering geometry in the wavenumber range of 100–3500 cm^−1^, by using an exciting radiation at 632.8 nm (He−Ne laser, power at the output ≈20 mW). The laser was focused onto the sample surface with a spot size of about 1 μm^2^ through the 80× objective (NA = 0.9) of a microprobe setup (Horiba-Jobin-Yvon LabRam HR800) consisting of a 80 cm focal length spectrograph using a 1800 grooves/mm grating and a charge-coupled device (CCD) detector cryogenically cooled by liquid nitrogen. The elastically scattered radiation was filtered by using a notch filter. In this configuration, the resolution was about 0.28 cm^−1^/pixel.

Fourier transform IR spectroscopy (FTIR) or attenuated total reflectance (ATR) absorption measurements were performed on dried samples, in the 600–4000 cm^−1^ wavenumber range and at room temperature. Spectra were recorded using a Bomem DA8 Fourier transform spectrometer, operating with a Globar source, in combination with a KBr beamsplitter, a DTGS/KBr detector. The powders were contained in Golden Gate diamond ATR system, just based on the ATR technique. The spectra were recorded in dry atmosphere, to avoid dirty contributions, with a resolution of 2 cm^−1^, automatically adding 100 repetitive scans to obtain a good signal-to-noise ratio and a high reproducibility. All the IR spectra were normalized for taking into account the effective number of absorbers.

The band deconvolution of the Raman and IR spectra in the 1600–1800 cm^−1^ wavenumber region was undertaken by using a multiple curve fitting procedure of the experimental profiles into Lorentzian functions. For each fitting session, multiple iterations were performed until a converging solution was reached by minimizing, in the meanwhile, the value of chi-square.

Thermogravimetric analyses were performed with a TA Instrument TGA2050 using the following heating program: equilibrate at 40 °C, ramp 10 °C/min to 700 °C in nitrogen. 40 mL/min and 60 mL/min are the gas flows applied in the balance and furnace section, respectively.

The ^1^H NMR spectra were recorded on a Bruker Avance 500 spectrometer operating at 500 MHz proton frequency equipped with a QNP four nuclei switchable probe. The experiment were carried out at 305 K. ^1^H NMR 1D experiments were performed using a standard residual water presaturation pulse sequence, SW = 5400 Hz, relaxation delay D1 = 4s, 32 K points for acquisition, 512 scans.

UV–vis absorption spectra were obtained with a Hewlett Packard UV–vis spectrophotometer model 845-3. A 10 mm path length rectangular quartz cells (Hellma) were employed in the 200–700 nm spectral range. Concentrations of the SF sample were adjust so that the extinction values did not exceed A = 1. SF was studied alone (1.0 × 10^−5^ M, pH 8.4) and in the presence of increasing amounts of branched β-CD polymer (3.3 × 10^−6^–1.3 × 10^−5^ M, pH 8.1–7.2).
